# Case series of four secondary mucormycosis infections in COVID-19 patients, the Netherlands, December 2020 to May 2021

**DOI:** 10.2807/1560-7917.ES.2021.26.23.2100510

**Published:** 2021-06-10

**Authors:** Jochem B Buil, Arthur R H van Zanten, Robbert G Bentvelsen, Tom A Rijpstra, Bram Goorhuis, Sanne van der Voort, Linda J. Wammes, Jeroen A Janson, Max Melchers, Moniek Heusinkveld, Willem J G Melchers, Ed J Kuijper, Paul E Verweij

**Affiliations:** 1 Department of Medical Microbiology, Radboud University Medical Center, and Radboudumc-CWZ Center of Expertise for Mycology, Nijmegen, the Netherlands; 2 Department of Intensive Care, Gelderse Vallei Hospital, Ede, the Netherlands; 3 Microvida Laboratory for Microbiology, Amphia Hospital, Breda, the Netherlands; 4 Department of Medical Microbiology, Leiden University Medical Center, Leiden, the Netherlands; 5 Department of Intensive Care, Amphia Hospital, Breda, the Netherlands; 6 Department of Infectious Diseases, Amsterdam University Medical Center, Amsterdam, the Netherlands; 7 Department of Intensive Care, Leiden University Medical Center, Leiden, the Netherlands; 8 Department of Medical Microbiology, Gelderse Vallei Hospital, Ede, the Netherlands; 9 Center for Infectious Disease Research, Diagnostics and Laboratory Surveillance National Institute for Public Health and the Environment (RIVM), Bilthoven, the Netherlands

**Keywords:** COVID-19, invasive mucormycosis

## Abstract

We describe four secondary fungal infections caused by Mucorales species in COVID-19 patients. Three COVID-19 associated mucormycosis (CAM) occurred in ICU, one outside ICU. All were men aged > 50 years, three died. Clinical presentations included pulmonary, rhino-orbital cerebral and disseminated infection. Infections occurred in patients with and without diabetes mellitus. CAM is an emerging disease and our observations underscore the need to be aware of invasive mucormycosis, including in COVID-19 patients without (poorly controlled) diabetes mellitus and outside ICU.

Ca 15% of patients with coronavirus disease (COVID-19) admitted to the hospital require mechanical ventilation in the intensive care unit (ICU) [1,2]. Secondary fungal infections were described in critically-ill ventilated COVID-19 patients in the initial reports from Wuhan, and subsequent studies from Europe reported variable frequencies of 3% to 33% of COVID-19 associated pulmonary aspergillosis (CAPA), with reports of azole resistant strains [3-7]. CAPA was associated with a 50% mortality rate and is mainly caused by *Aspergillus fumigatus* [8]. Recently, secondary fungal infections caused by Mucorales in critically ill COVID-19 patients were reported from India, with mortality rates ranging between 24.3% and 81% depending on the clinical manifestation [9,10].

We describe a series of four cases of secondary infection with Mucorales in real-time PCR confirmed COVID-19 patients in the Netherlands diagnosed between December 2020 and May 2021, of which three developed the infection during ICU admission and three died.

## Case series

Case 1 is a man in his mid-60s who was admitted to the ICU of a general hospital 8 days after the first COVID-19 symptoms. He had no underlying diseases and received a single dose of tocilizumab and dexamethasone over 10 days starting on day 1 of ICU admission. The patient was clinically stable until he developed bilateral consolidations with no typical radiologic fungal infection signs on computed tomography (CT) on day 9 at ICU. The same day oropharynx and sputum cultures were positive with *Rhizopus microsporus* and a bronchoscopy was performed, with *R. microsporus* growing in a BAL culture*.* Treatment with liposomal amphotericin B was initiated on day 13, and after clinical deterioration and worsening consolidations on chest CT posaconazole was added on day 19. The patient is currently continuing antifungal treatment, while receiving mechanical ventilation in the ICU.

Case 2 is a man in his late 50s who was admitted to the ICU of a University Medical Centre 6 days after onset of COVID-19 symptoms. The patient had no underlying diseases and was treated with dexamethasone from day 1 of ICU admission and received one dose of tocilizumab. Voriconazole was started because of positive *A. fumigatus* sputum and BAL cultures on day 6 in ICU, but a sputum culture obtained on day 9 was also positive with *Lichtheimia ramosa*. CT scan showed pulmonary cavities and a reversed halo-sign. Repeat sputum and cultures from three BALs were subsequently positive with *L. ramosa*, and voriconazole therapy was changed to liposomal amphotericin B and posaconazole. The clinical condition deteriorated, and the patient died on day 21 of ICU admission. An autopsy was not performed.

Case 3 is a man in his late 60s with known chronic lymphocytic leukaemia that had been stable for 8 years. The patient was also diagnosed with diabetes mellitus and obesity. He had COVID-19, was admitted to the ICU of a general hospital for mechanical ventilation because of progressive respiratory failure, and was treated from day 1 on ICU with dexamethasone, later changed to prednisone and a single dose of tocilizumab on day 1. On day 10 of ICU admission, respiratory deterioration and acute-onset kidney failure occurred. Treatment with voriconazole and micafungin was initiated due to positive sputum cultures with *A. fumigatus*, but on day 21, *R. microsporus* was cultured from sputum. A subsequent lung biopsy was negative, but antifungal therapy was changed to isavuconazole, and later liposomal amphotericin B was added. Subsequent CT scans on day 30, showed no evidence for sinusitis and cerebral lesions, but indicated progression of pulmonary lesions and dissemination to the kidneys, and microscopy showed non-septate hyphae in the patient’s urine. The patient died and disseminated mucormycosis was confirmed at autopsy.

Case 4 is a man in his early 70s who had been treated for COVID-19, including systemic corticosteroids, in a hospital abroad. Within a month after discharge, he was re-admitted in the same country for treatment of a cerebrovascular stroke causing left-sided paralysis. During this admission, his known diabetes mellitus was poorly controlled. Shortly after returning to the Netherlands, he was referred to a University Medical Centre with fever and swelling of his right eye, with loss of vision, caused by extensive sinusitis with intracranial necrosis and infarction. Orbital pus was positive for *R. arrhizus*. Surgical debridement and treatment with liposomal amphotericin B, isavuconazole and interferon-γ did not successfully contain the infection, and the patient died.

The table provides an overview of the cases and the treatment received and clinical outcome.

**Table:** Characteristics of four cases with COVID-19 associated mucormycosis, the Netherlands, December 2020 to May 2021

Case	Sex / age	Underlying disease/condition	CAM diagnosis (days after COVID-19 diagnosis)	Clinical presentation	EORTC/MSGERC classification [16]	Evidence for CAPA (days after ICU admission)	COVID-19 treatment (days after ICU admission)	Antifungal therapy (days after ICU admission)	Outcome (days after COVID-19 diagnosis)
1	M / mid-60s	None	22	Respiratory failure	Probable^b^	No	Tz 600 mg (single dose)(1); Dx 6 mg (x1) (1–10); Prednisolon 60 mg (x1) (10–12)	VCZ (0–13); L-AmB (13-ongoing); POS (19-ongoing)	Alive
2	M / late 50s	None	17	Respiratory failure	Probable^b^	*Aspergillus fumigatus* in sputum and BAL (4)	Tz 600 mg (single dose)(1); Dx 6 mg (x1) (1–10)	VCZ (7–12); L-AmB (12–23); POS (13–23)	Died (27)
3	M / late 60s	CLL, DM, obesity	35	Respiratory failure, acute onset kidney failure	Proven	*A. fumigatus* in BAL culture, positive BAL-GM, tracheal plaques at bronchoscopy (9)	Tz 800 mg (single dose)(1); Dx 6 mg (x1) (-3-7)	VCZ + AFG (9–21); ISA (21- 24); VCZ (24- 30); ISA (30- 35); ISA + L-AmB + INF- γ (35–43); AmB bladder irrigation (39- 43)	Died (46)
4	M / early-70s	DM, steroid therapy	± 88^a^	Extensive sinusitis	Probable	No	Dx (treatment abroad, dose and duration unknown)	L-AmB + ISA + INF- γ for 7 weeks	Died (129)

BAL: bronchoalveolar lavage; CAM: COVID-19 associated mucormycosis; CAPA: COVID-19 associated pulmonary aspergillosis; CLL: chronic lymphocytic leukaemia; COVID-19: coronavirus disease; d: day; DM: diabetes mellitus; Dx: dexamethasone; EORTC/MSGERC: European Organization for Research and Treatment of Cancer and the Mycoses Study Group Education and Research Consortium; GM: galactomannan; ICU: intensive care unit; INF-γ: interferon-γ; ISA: isavuconazole; L-AmB: liposomal amphotericin B; POS: posaconazole; M: male; Tz: tocilizumab; VCZ: voriconazole.

^a^ First CAM symptoms on day 45, but diagnosis was made on day 88.

^b^ Cases did not have EORTC/MSGERC host factors.

## Discussion

Mucormycosis is a rare invasive fungal disease often seen in immunocompromised individuals, in patients with diabetic ketoacidosis and in patients with concomitant use of steroids [11]. Cases of mucormycosis are now being reported in patients with severe COVID-19, associated with uncontrolled diabetes mellitus and systemic corticosteroid therapy [9]. Numerous cases of COVID-19 associated mucormycosis (CAM) are reported from India, which is known to have a high burden of (poorly regulated) diabetes mellitus [9,12]. However, a recent cohort of 38 CAM cases was reported from outside India, including cases from Europe, the Middle East, North and South America, with an estimated prevalence of 0.3% to 0.8% [10]. Common characteristics of patients with CAM included the presence of a risk factor in 95% of patients, mainly diabetes mellitus (82.5%). In most patients, the diabetes mellitus was poorly or uncontrolled (80.3%), and 75% of patients received systemic corticosteroids. CAM presented mainly as rhino-orbital cerebral mucormycosis, especially in patients with diabetes mellitus, and pulmonary infection, while disseminated disease was uncommon [10]. Furthermore, due to surgical interventions in patients with rhino-orbital cerebral mucormycosis, loss of vision was common in survivors [10].

We report four CAM cases, one confirmed, three probable, all of which occurred in men aged between in their late 50s and mid-70s, receiving corticosteroids, while only two had underlying diseases. CAM was characterised by a variety of clinical presentations, including pulmonary, rhino-orbital cerebral and disseminated infection. CAM occurred in COVID-19 cases admitted to the ICU and in one non-ICU case, cases without diabetes mellitus, and cases with concomitant CAPA. Furthermore, cases were observed both in academic hospitals as well as general hospitals. CT-scan was unhelpful in most cases to diagnose CAM due to extensive COVID-19 lesions, although in one case a reversed halo-sign was observed. In the three ICU cases, Mucorales were first cultured in sputum, routinely collected in Dutch ICUs from patients receiving selective decontamination of the gastrointestinal tract, who are screened for Gram-negative bacteria and yeasts. In all cases, subsequent diagnostic interventions (2 x BAL and lung biopsy) were performed to confirm CAM diagnosis. CAM diagnosis may be difficult due to the lack of a specific biomarker and the limited availability of molecular detection tests of Mucorales in clinical microbiology laboratories. Despite appropriate antifungal therapy, three of four cases died, while one is currently continuing antifungal treatment in the ICU.

Three of the cases described here had received the anti-interleukin (IL)-6 receptor monoclonal antibody tocilizumab, but the role of immunotherapy as risk factor for developing secondary invasive fungal infections, including mucormycosis, remains unclear [13]. Reducing tissue damage through dampening hyperimmune reactivity, such as cytokine storm syndrome, may lower the risk for invasive mycoses, while at the same time, immunomodulation interventions might increase host susceptibility to invasive fungal disease [14].

Our observations underscore the need to be aware of invasive mucormycosis developing in COVID-19 patients, especially when receiving corticosteroids, including patients without (poorly controlled) diabetes mellitus and outside the ICU. Positive cultures with Mucorales from the sputum of other respiratory tract samples should be considered relevant and prompt a diagnostic workup, also when cultures are recovered in combination with *Aspergillus*. An aggressive approach is recommended when invasive mucormycosis is diagnosed, including early surgery and targeted antifungal treatment [15].
